# *Akkermansia muciniphila* promotes testosterone‐mediated hair growth inhibition in mice

**DOI:** 10.1096/fba.2023-00056

**Published:** 2023-10-25

**Authors:** Eunyoung Lee, Daedong Kim, Hyo‐Deok Seo, Jeong‐Hoon Hahm, Jae‐Gu Seo, Sang‐Nam Lee, Do‐Hak Kim, Jiyun Ahn, Chang Hwa Jung

**Affiliations:** ^1^ Aging and Metabolism Research Group Korea Food Research Institute Wanju‐gun Jeollabuk‐do Republic of Korea; ^2^ Department of Food Biotechnology University of Science and Technology Wanju‐gun Jeollabuk‐do Republic of Korea; ^3^ R&D center Enterobiome Inc. Goyang‐si Republic of Korea

**Keywords:** *Akkermansia muciniphila*, growth factors, hair growth, testosterone, β‐Catenin

## Abstract

The beneficial effects of *Akkermansia muciniphila* (*Akk*) on gut health and inflammation reduction have been demonstrated; however, scientific evidence of hair growth enhancement by *Akk* has not been reported. Therefore, this study was undertaken to investigate the effect of *Akk* on improving testosterone‐mediated hair growth inhibition. Hair growth inhibition was induced through subcutaneous injection of testosterone into the shaved dorsal skin of C57BL/6 male mice. Live and pasteurized *Akk* were orally administered at a concentration of 1 × 10^8^ colony‐forming unit. After 5 weeks, hair length and skin tissues were analyzed. The live and pasteurized *Akk* significantly stimulated hair growth, countering the inhibitory effect of testosterone compared to the testosterone‐alone group. Hematoxylin and eosin staining revealed a significant increase in hair follicle size in the *Akk*‐treated group. An increase in β‐catenin levels, which are associated with hair growth and cell cycle progression, was also observed. Moreover, the *Akk*‐treated group exhibited increased levels of fibroblast growth factors, including *Fgf7*, *Igf1*, *Fgf7*, *Fgf10*, and *Fgf21*. However, no significant difference was observed between the live and pasteurized *Akk* groups. These results underscore the potential of live and pasteurized *Akk* in improving testosterone‐mediated hair growth inhibition.

## Graphical abstract

This study investigated whether *Akkermansia muciniphila* (*Akk*) improves testosterone‐induced hair growth inhibition. Live and pasteurized *Akk* was orally administered, and after 5 weeks, hair length and skin tissues were analyzed. The live and pasteurized *Akk* significantly stimulated hair growth, countering the inhibitory effect of testosterone compared to the testosterone‐alone group.

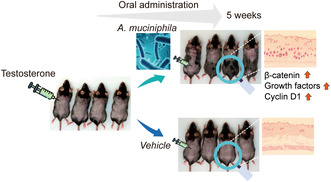

## 1 INTRODUCTION

*Akkermansia muciniphila* (*Akk*), a mucin‐degrading bacterium found in the mucus layer of the human gut, is a potential probiotic as it helps regulate the immune system and reduce inflammation.^1^, ^2^ However, despite growing interest in *Akk*, its precise mechanisms of action and potential human health applications are yet to be fully elucidated. Several studies have examined the potential benefits of *Akk* as an oral supplement, particularly in the form of capsules and beverages. For example, live *Akk* bacteria significantly reduced insulin resistance, total cholesterol, low‐density lipoprotein cholesterol, and body weight compared to the placebo group.^3^, ^4^ *Akk* improves gut barrier function and reduces autoimmune and chronic inflammatory diseases.^5^ In addition, daily administration of *Akk* prevents age‐related decline in colonic mucous layer thickness, thereby attenuating inflammation and immune‐related processes in old age, suggesting that *Akk* may contribute to the promotion of healthy aging.^6^ These results suggest that *Akk* may have potential health benefits. However, further research is required to fully understand its effects on human health and determine how best it can be incorporated into diets.

Although there is currently no documented evidence regarding the positive effects of *Akk* on hair health and loss, it holds promise in this regard as it promotes a balanced gut‐immune interaction and reduces intestinal and systemic inflammation. Studies have reported that the gut microbiome may influence conditions such as alopecia areata by regulating inflammation and the immune system.^2^ For instance, the overgrowth of *Lactobacillus murinus* impairs intestinal metabolic function and causes alopecia.^7^ However, direct investigations into the relationship between the gut microbiome and androgenetic alopecia are lacking. A cohort study revealed that the scalp and intestinal microbiota of patients with male pattern baldness differed significantly from those of the normal group.^8^ Given the impact of the gut microbiome on various health conditions, the link between the gut microbiome and androgenetic alopecia merits further exploration. Therefore, the efficacy evaluation of *Akk* in male pattern baldness holds value as a preliminary study.

Hair growth is a complex process that depends on various factors, including genetics, hormone levels, and overall health.^9^ Testosterone contributes to hair loss in men and women; therefore, inhibiting its effects can promote hair growth. Oral medications for androgenetic alopecia treatment, which block the conversion of testosterone to dihydrotestosterone, include finasteride and dutasteride.^10^ Although not all patients experience side effects from these drugs, sexual side effects, breast tenderness or enlargement, skin rash or itching, swelling of the hands or feet, headaches, dizziness, depression, and anxiety may be expected.^10^ Therefore, active studies are being conducted to develop hair loss treatments that do not induce side effects. Stem cell therapies have been proposed^11^; however, further research is required to ensure their safety. To explore new avenues for hair growth improvement, beneficial human gut microbes have been proposed as new targets. However, to date, there is no scientific evidence that *Akk* promotes hair growth. Therefore, in this study, we aimed to examine how Akk could enhance hair growth in the presence of testosterone‐induced inhibition, which was induced by administering subcutaneous testosterone injections into the shaved dorsal skin of C57BL/6 male mice.

## 2 MATERIALS AND METHODS

### 2.1 Culture and pasteurization of *Akkermansia muciniphila*

*Akk* (EB‐AMDK19, KCTC13761BP) was isolated from the fecal samples of healthy Korean subjects^12^ and anaerobically cultured in a soy‐peptone‐based medium containing 20 g/L soy‐peptone, 10 g/L yeast extract, 2.5 g/L K_2_HPO_4_, 5 g/L N‐acetyl‐D‐glucosamine, 2.5 g/L D‐fructose, 5 g/L D‐lactose, 8 g/L L‐aspartic acid, 0.1 mg/L cyanocobalamin, and 0.5 g/L L‐cysteine hydrochloride at 37°C. To prepare the pasteurized form, cultures were inactivated by pasteurization at 70°C for 30 min. The live and pasteurized cells were then obtained by centrifugation (12,000 *g*, 5 min, 4°C) and lyophilized under anaerobic conditions. Lyophilized powders were immediately frozen and stored at −80°C until use. Ethical approval was granted at the Public Institution Bioethics Committee under the Ministry of Health and Welfare, Korea (approved number: P01‐201705‐31‐002). Before oral gavage administration, the freeze‐dried powders were resuspended in an appropriate volume of anaerobic phosphate‐buffered saline (PBS) to an end concentration of 1 × 10^8^ colony‐forming units (CFU)/100 μL. The number of live and nonviable cells was counted using the spread plate and Petroff–Hausser counting methods, respectively.

### 2.2 Animal experiments

After shaving the backs of seven‐week‐old C57BL/6 male mice adapted to the breeding room environment, they were separated into four groups of eight mice each. All the groups were fed a chow diet. Testosterone (0.5 mg/day) was injected subcutaneously to inhibit hair growth, finasteride (0.5 mg/kg/day) was orally administered to the positive control group, and live and pasteurized *Akk* were orally administered to the experimental groups at a concentration of 1 × 10^8^ CFU. The experiment was conducted for 5 weeks by subcutaneous injection and oral administration five times a week. Hair growth was observed by photographing the hair of the experimental animals once a week, and hair length, organs, and skin tissues were secured through an autopsy after the experiment was completed. All animal experiments were approved by the Institutional Animal Care and Use Committee of the Korea Food Research Institute (KFRI‐M‐21060).

### 2.3 Hair analysis

The degree of hair growth was observed by photographing each group weekly, and the length of hair obtained from the dissection was analyzed microscopically.

### 2.4 H&E stain

Mouse skin tissues were fixed in formaldehyde, embedded in paraffin, and stained with hematoxylin and eosin (H&E) solution. Hair follicles were observed at 200× magnification using a microscope (Olympus).

### 2.5 Immunoblotting

Proteins were extracted from the skin tissue using radioimmunoprecipitation assay lysis buffer (Thermo Scientific). Protein quantification samples were electrophoresed on sodium dodecyl sulfate‐polyacrylamide gels, transferred to polyvinylidene fluoride membranes (Trans‐Blot, Bio‐Rad Laboratories), blocked with 5% skim milk, incubated with primary antibody, washed with tris‐buffered saline with tween (TBST), incubated with secondary antibody, and washed with TBST; the target protein was detected using an enhanced chemiluminescence solution. Antibodies against β‐actin and cyclin D1 (sc‐8396) were purchased from Santa Cruz Biotechnology. Antibodies against phosphorylated protein kinase B (p‐Akt) (4060 s), Akt (9272 s), and β‐catenin (9582 s) were purchased from Cell Signaling Technology.

### 2.6 Quantitative RT‐PCR

Total RNA from skin tissues was extracted using RNeasy Mini Kit (Qiagen). The cDNA was synthesized using the ReverTra Ace® qPCR RT kit (Toyobo), and quantitative RT‐qPCR was performed using SYBR Green real‐time PCR Master Mix (Toyobo). The primer sequence of the growth factor genes was analyzed with reference to previous papers.^13^ Fibroblast growth factor 7 (*Fgf‐7*): *Forward* 5′‐AGACTGTTCTGTCGCACC‐3′ and reverse 5′‐CCGCTGTGTGTCCATTTAG‐3′; *Fgf‐10*: *Forward* 5′‐TGTCCGCTGGAGAAGGCTGTTC‐3′ and reverse 5′‐CTATGTTTGGATCGTCAT GG‐3′; *Fgf‐21*: *Forward* 5′‐CTATGTTTGGATCGTCATGG‐3′ and reverse 5′‐CGGCCCTGTAAAGGCTCT‐3′; Insulin‐like growth factor 1 (*Igf‐1*): *Forward* 5′‐TCAACAAGCCCACAGGGTAT‐3′ and reverse 5′‐ACTCGTGCAGAGCAAAGGAT‐3′; vascular endothelial growth factor (*Vegf*); *Forward* 5′‐TCTTCAAGCCATCCTGTGTG‐3′ and reverse 5′‐ GCGAGTCTGTGTTTTTGCAG‐3′; *Gapdh*: *Forward* 5′‐TGGATTTGGACGCATTGGTC‐3′ and reverse 5′‐TTTGCACTGGTACGTGTTGAT‐3′.

### 2.7 Statistical analysis

Statistical analyses were conducted using one‐way analysis of variance with Tukey's test for between‐group multiple comparison analysis using GraphPad Prism Version 9.0 software (GraphPad Software Inc.). A *p*‐value < 0.05 was considered statistically significant, and data are expressed as mean ± standard error of the mean.

## 3 RESULTS

### 3.1 *Akk* improved testosterone‐induced hair growth inhibition

**FIGURE 1:**
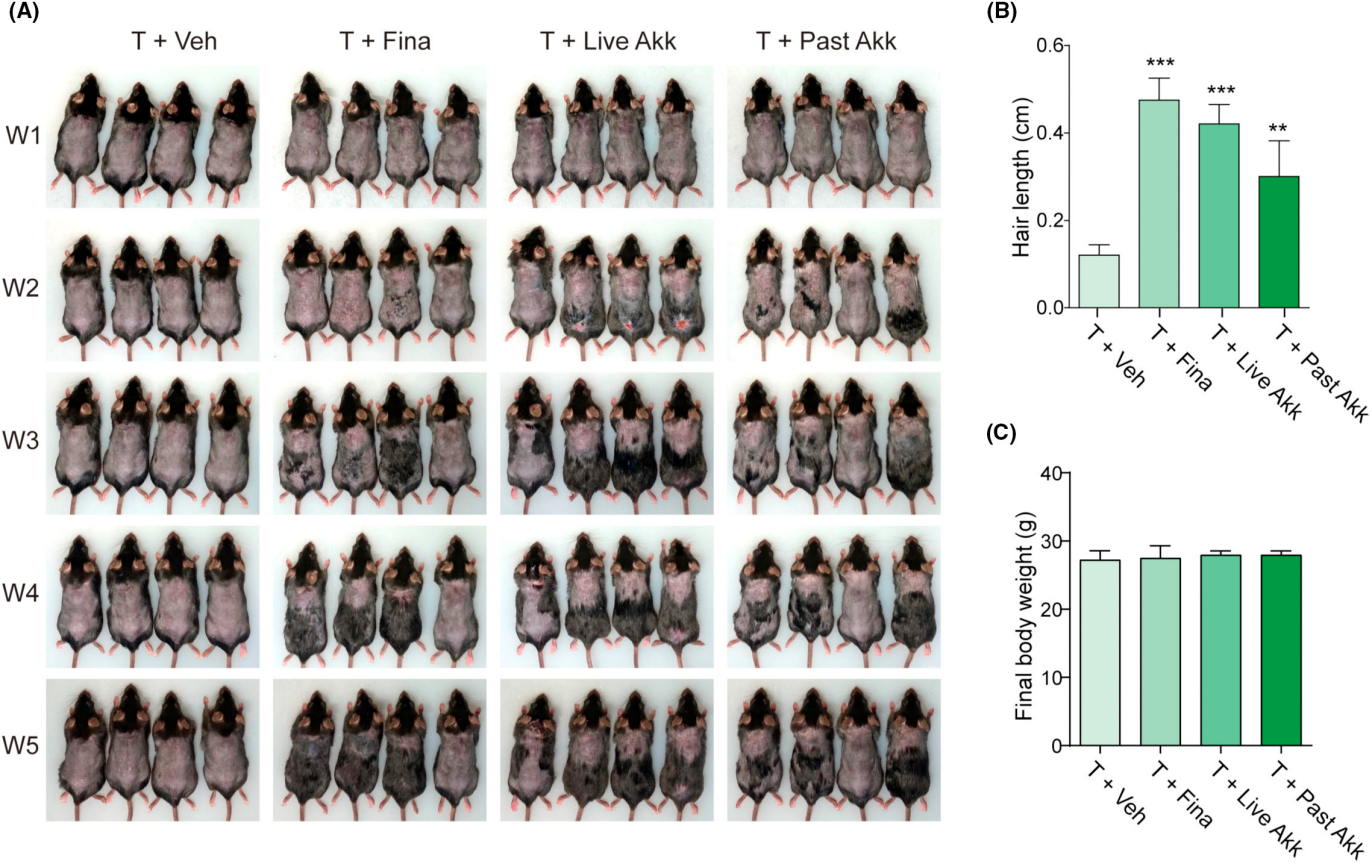
*Akk* improved testosterone‐induced hair growth inhibition in C57BL/6 mice. (A) Hair phenotype of live and pasteurized *Akkermansia muciniphila (Akk)* on hair growth (*n* = 8 mice/group). Photographs were obtained once a week. Hair length (B) and final body weight (C) were measured at 5 weeks. **p < 0.01, ***p < 0.001, compared to the testosterone (T + Veh) group.

Subcutaneous injection of testosterone effectively inhibited hair growth in shaved mice (Figure 1A). Oral administration of live and pasteurized *Akk* initiated hair growth at 2 weeks and induced significant hair growth compared to the testosterone‐only group at the end of the experiment (5 weeks; Figure 1B). Although the live *Akk* group exhibited a pattern of increased hair length compared to the pasteurized *Akk* group, the difference was not significant. The hair growth efficacy of *Akk* was comparable to that of finasteride, which was used as the positive control drug. No significant change in body weight was observed in any of the experimental groups (Figure 1C).

### 3.2 *Akk* increased hair follicles in the skin

**FIGURE 2:**
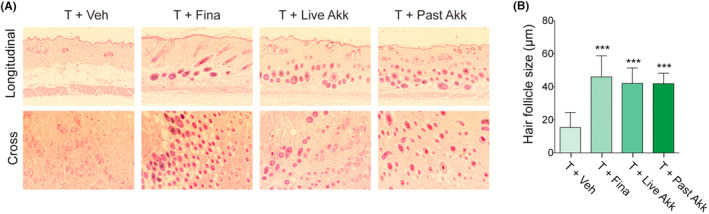
*Akk increased hair follicles*. (A) Histological morphology was observed using hematoxylin & eosin (H&E) staining of mouse skin after 5 weeks (*n* = 4 mice/group). (B) Quantification of hair follicle sizes. ***p < 0.001, compared to the testosterone (T + Veh) group.

An increase in hair follicle density indicates the transition of hair growth from the telogen to the anagen phase.^14^ H&E staining was performed to investigate the progression of hair follicles during the hair cycle. In the longitudinal and cross sections, hair follicles were observed more clearly in the *Akk*‐treated groups than in the testosterone‐only‐treated mice (Figure 2A). The number of hair follicles was considerably increased in the *Akk*‐treated mice compared to that in the testosterone‐only‐treated mice at week five (Figure 2B). These results suggest that *Akk* promoted hair growth by inducing the anagen phase of the hair follicles. However, no significant difference was observed between live and pasteurized *Akk* treatment groups.

### 3.3 *Akk* stimulated hair growth‐related factors

**FIGURE 3:**
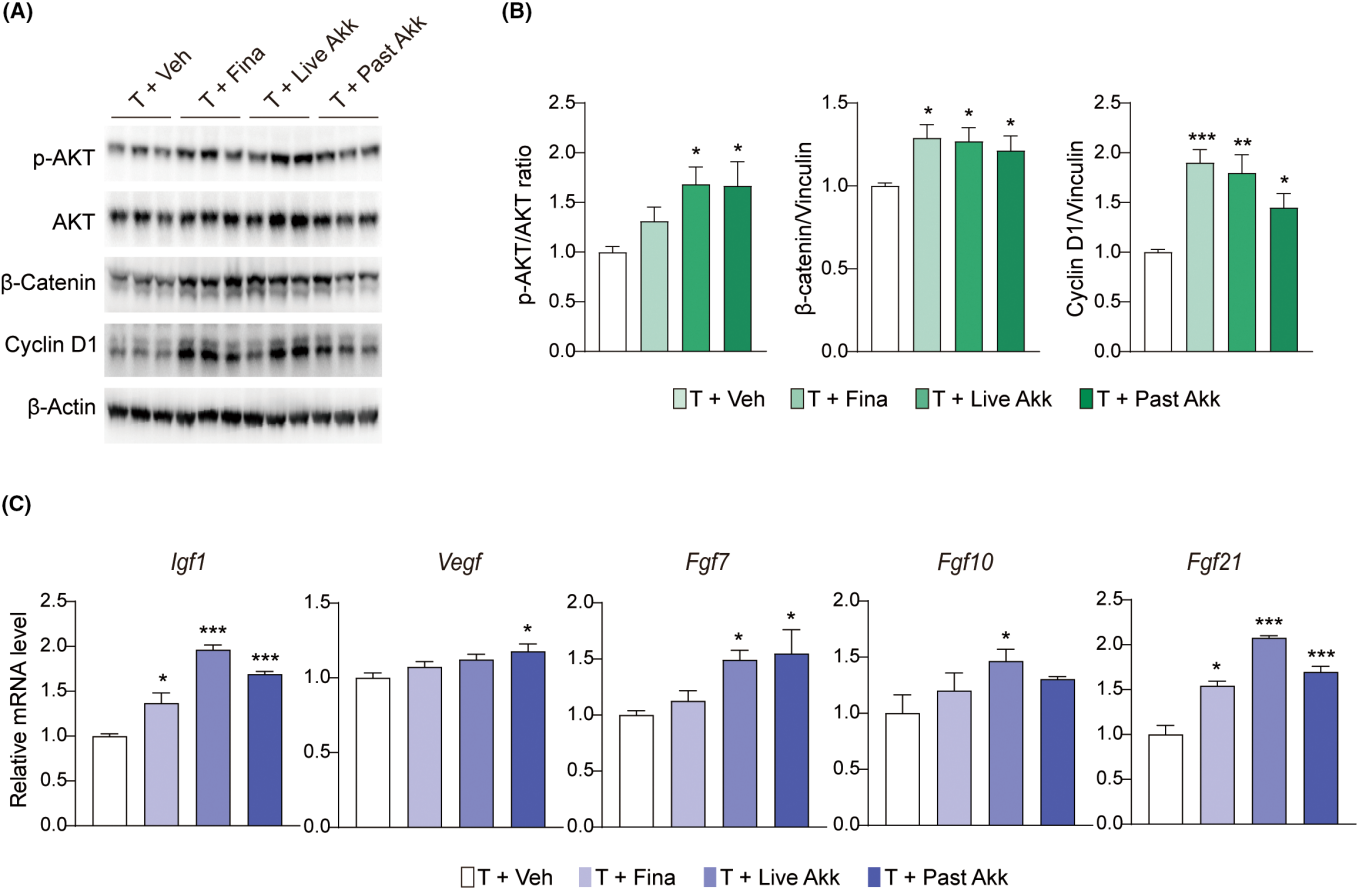
*Akk increased testosterone‐induced hair growth factors inhibition in C57BL/6 mice*. (A) Immunoblot analysis of β‐catenin pathway in the skin. (B) Relative protein levels were quantitated using ImageJ software. (C) mRNA levels of growth factor in the skin. *p < 0.05, **p < 0.01, compared to the testosterone (T + Veh) group.

AKT plays a critical role in hair growth regulation by promoting the survival and proliferation of hair follicle cells.^15^ Live and pasteurized *Akk* increased the testosterone‐induced inhibition of p‐AKT and exerted a more pronounced increase in AKT phosphorylation than finasteride (Figure 3A,B). Live and pasteurized *Akk* increased β‐catenin levels, a key regulatory protein in hair growth,^16^ although significant differences were not observed between the live and pasteurized *Akk* groups. *Akk* also increased the expression levels of cyclin D1, which is crucial in regulating cell cycle progression.^17^ Furthermore, we observed a significant increase in the family members of the fibroblast growth factor, including *Fgf7*, *Igf1*, *Fgf7*, *Fgf10*, and *Fgf21* in the *Akk*‐treated groups (Figure 3C). Collectively, *Akk* appears to influence hair growth by regulating cell proliferation through β‐catenin activation, cell cycle progression, and growth factor expressions.

## 4 DISCUSSION

*Akkermansia muciniphila* regulates immunity, metabolism, and inflammation by maintaining a healthy intestinal barrier.^18^, ^19^ However, studies on its effect on hair health are yet to be reported. In this study, we aimed to investigate the potential positive effects of *Akk* on not only alopecia areata but also hormonal hair suppression. By employing a mouse model to simulate testosterone‐induced hair growth inhibition, we found that *Akk* holds the potential to improve testosterone‐induced hair growth inhibition. This effect aligned with that of finasteride, a widely recognized treatment for hair loss,^20^ suggesting that *Akk* can potentially suppress hormonal hair loss. However, further clinical studies are warranted to validate its efficacy.

After the inactivation of bacteria, mainly through heat treatment, dead cells can release bacterial components with key immunomodulating effects and antagonizing properties against pathogens. Different bacterial components, such as lipoteichoic acids, peptidoglycans, or exopolysaccharides, have been proposed to play a central role in these properties with formulations containing heat‐killed bacteria.^21^, ^22^, ^23^ Notably, the outer membrane protein Amuc 1100 of *Akk* has demonstrated stability at pasteurization temperature. It has shown efficacy in ameliorating *Porphyromonas gingivalis*‐induced alveolar bone loss and periodontal inflammation in mice by promoting macrophage polarization toward the anti‐inflammatory M2 phenotype and cytokine interleukin‐10 expression.^24^ Moreover, Kasuya et al.^25^ have reported that increased infiltration of M2 macrophage into the skin wound tissue of mice could promote hair follicle regeneration through the production of growth factors such as Igf1 and Fgf2.

Several biomarkers associated with the growth of dermal papilla cells, such as AKT, β‐catenin, and cyclin D1, were evaluated in this study. AKT signaling is involved in the anagen phase, which is the active growth phase of hair follicle cells. During the anagen phase, AKT is activated by various growth factors and signaling molecules, including IGF‐1, FGF7, and wingless/integrated (Wnt) signaling.^16^ AKT activation promotes the survival and proliferation of hair follicle cells and regulates the differentiation of hair follicle stem cells into hair shaft‐producing cells.^26^ β‐catenin is a component of the Wnt signaling pathway, which regulates various cellular processes, including cell proliferation, differentiation, and survival.^27^ During the anagen phase, β‐catenin is highly expressed in the hair follicle bulb and matrix cells. β‐catenin promotes the proliferation and differentiation of hair follicle stem cells and hair shaft‐producing cells by activating the expression of various target genes involved in hair growth.^27^ In summary, β‐catenin and AKT play a crucial role in hair growth by regulating the hair growth cycle. Therefore, *Akk* may be involved in hair growth via these signaling pathways.

It is necessary to understand *Akk*‐induced changes in the gut microbiome by establishing the elemental microbial composition of animals prior to experiments and regularly monitoring microbiome changes in fecal samples after oral *Akk* administration. Based on the changes observed in the microbiome, additional experiments can be performed to elucidate the mechanisms of hair growth and gut environment. For example, microbial metabolites or the expression of genes involved in host–microbe interactions may be analyzed. Additionally, *Akk* intake and frequency require further investigation. Furthermore, the analysis of stem cells or dermal papilla cells isolated from tissues and biomarkers related to hair growth may improve the understanding of the action mechanisms of *Akk*.

Taken together, these results suggest that *Akk* improves testosterone‐induced hair growth inhibition. In addition, it can be inferred that *Akk* increased the expression of hair growth factors by regulating AKT and β‐catenin signaling pathways associated with hair growth. Notably, no difference was observed between the live and pasteurized *Akk* groups.

## AUTHOR CONTRIBUTIONS

C.‐H. Jung designed the experiment and drafted the manuscript; E. Lee and H.‐D. Seo performed animal experiments; J.‐H. Hahm and J. Ahn performed data curation. J.‐G. Seo, S.‐N. Lee, and D.‐H. Kim prepared *Akkermansia muciniphila*. All authors contributed to the discussion of results and manuscript revision.

## CONFLICT OF INTEREST STATEMENT

The authors declare no conflict of interest.

## Data Availability

The data that support the findings of this study are available on request from the corresponding author. The data are not publicly available due to privacy, legal, or ethical restrictions.
